# Renal tumors and second primary pancreatic tumors: a relationship with clinical impact?

**DOI:** 10.1186/1754-9493-6-18

**Published:** 2012-08-08

**Authors:** Sascha A Müller, Sascha Pahernik, Ulf Hinz, David J Martin, Moritz N Wente, Thilo Hackert, Christine Leowardi, Axel Haferkamp, Markus W Büchler, Bruno M Schmied

**Affiliations:** 1 Department of General, Visceral and Transplant Surgery, University of Heidelberg, Heidelberg, Germany; 2 Department of Urology, University of Heidelberg, Heidelberg, Germany; 3 Unit for Documentation and Statistics, Department of Surgery, University of Heidelberg, Heidelberg, Germany; 4 Department of Surgery, Kantonsspital St. Gallen, Rorschacherstrasse 95, St.Gallen, CH – 9007, Switzerland

**Keywords:** Pancreatic carcinoma, Renal cell carcinoma, Double primary carcinoma, Synchronous and metachronous cancers

## Abstract

**Background:**

The occurrence of synchronous or metachronous renal cell carcinoma and pancreatic tumors has been described only in a few cases in the scientific literature. The study of double primary cancers is important because it might provide understanding of a shared genetic basis of different solid tumors and to detect patients at risk for secondary malignancy.

**Methods:**

In a combined analysis of patient registries from University Departments of Urology and Visceral Surgery, 1178 patients with pancreatic tumors and 518 patients with renal cell carcinoma treated between 2001 and 2008 were evaluated,

**Results:**

Overall 16 patients with renal cancer and synchronous (n = 6) or metachronous (n = 10) primary pancreatic tumors were detected. The median survival of all patients was 12.6 months, for the patients with synchronous resections 25.7 months and for the patients with metachronous resections 12.2 months, respectively.

**Conclusions:**

The association between these two etiologies of malignancy demands more detailed epidemiological and molecular investigation. Clinical outcomes would support a resection as a recommended clinically valid option.

## Background

Recent improvements in the prognosis of cancer patients have led to an increase in the incidence of second primary cancers, and the frequency of multiple primary malignant tumors is expected to increase as the population ages [1]. Moreover, the widespread application of computertomography (CT) and ultrasound (US) for other indications has led to increased detection of renal and pancreatic tumors as an incidental finding [2]. These tumors are typically smaller than those that produce symptoms and are more likely to be resectable. The frequency of multiple primary tumors among all cases of malignancy has been reported as 1 to 3% [3]. The frequency of pancreatic cancer in association with cancer of other organs is estimated to range from 1% to as high as 20% [4], with malignancies predominately of the stomach, colon, thyroid, and genitourinary tract [5]. Second malignancies reported to be associated with renal cell carcinoma (RCC) include Non-Hodgkin’s lymphoma, multiple myeloma, chronic lymphatic leukaemia, melanoma and cancers of the bladder, prostate, breast, rectum, and lung with an incidence that varies from 5 to 27% [6,7]. There has only been infrequent reporting of synchronous or metachronous tumors of the pancreas and the kidney [5,7,8].

RCC, originating in the renal cortex and accounting for 80% to 85% of malignant kidney tumors, represents 2% to 3% of all cancers [9], with the highest incidence occurring in more developed countries. Rates of RCC vary internationally more than 10-fold, suggesting a strong role for exogenous risk factors, in addition to possible roles of geographic differences in genetic susceptibility and diagnostic variability. Several risk factors have been identified including increased age, male sex, smoking, obesity, long-term dialysis, and several genetic syndromes including familial clear cell carcinoma, von Hippel-Lindau disease (VHL) and tuberose sclerosis [10,11]. Pancreatic manifestations of the rare autosomal dominant VHL disease include simple cysts, diffuse cystic changes, cystadenomas and tumors [12]. Approximately 35% to 45% of patients with VHL develop kidney cancers that are of clear cell histology, often bilateral and/or multifocal and is a major cause of death among these patients [12]. Cigarette smoking is the most consistently established causal risk factor for RCC and doubles the likelihood of RCC and contributes to as many as one third of all cases [13]. Second primary malignancies associated with RCC include those of urinary bladder, prostate, rectal, and lung cancer, as well as non-Hodgkin’s lymphoma and melanoma [7].

Pancreatic ductal adenocarcinoma (PDAC) comprises 2% of all cancer diagnoses and is a highly malignant carcinoma, making it the forth leading cause of cancer-related death [14]. Unfortunately, due to the late presentation of symptoms, only 10% to 20% of patients are candidates for surgical resection, which remains the only viable chance for cure [15]. The precise causes of pancreatic cancer have not yet been determined, but research indicates that certain risk factors may be associated with an increased probability of developing pancreatic cancer. These factors include smoking, age, race, family history, obesity, chronic pancreatitis, environmental factors, and genetic predisposition [16,17]. Ten per cent of all PDAC cases are related to genetic disorders, e.g. BRCA1 and BRCA2 gene mutations, hereditary non-polyposis colorectal cancer (HNPCC, Lynch syndrome) and familial atypical mole-malignant melanoma (FAMMM) [18-20].

To the best of our knowledge, only few cases with synchronous or metachronous occurrence of both tumors have been reported in the literature. In the present study data from two registries (Department of Surgery and Department of Urology) were screened. We examined for the first time surgical outcome, and prognosis of coexisting primary renal and pancreatic tumors in the same patient observed and treated either synchronously or metachronously.

## Methods

This study prospectively analyzed 1178 patients who underwent exploration or resection for PDAC or pancreatic cystic tumors at the Department of Surgery, from October 2001 through March 2008 and 518 patients with RCC treated during the same time period at the Departement of Urology. Among these patients, we analyzed those who had undergone a surgical procedure for pancreatic tumors and simultaneous or preceding surgery for synchronous or metachronous renal tumors. Synchronous second primary tumors were defined as tumors diagnosed at the same time or within 12 months before the diagnosis of the first primary tumor. If both tumors were present at the same time, the oncological resection was performed interdisciplinary through a standard median laparotomy. Metachronous pancreatic tumors occurred more than 12 months after the primary renal tumor. Patients with ampullary adenocarcinomas, distal bile-duct carcinomas, and other malignancies were excluded from the study. PDAC and neoplastic cystic pancreatic tumors were both defined as pancreatic tumors in the current study. This study identified a total of 16 patients with double primary tumors. Sixteen partial or total nephrectomies and exploration or resection for pancreatic tumors were perfomed within 0 to 205 months of diagnosis. The synchronous second primary tumors were confirmed by pathological diagnosis after resection. The demographic and clinical variables, including age, sex, operative procedure, morbidity, mortality and hospital course were collected. All patients were regularly followed in the outpatient clinic, or the patients’s primary physician was personally contacted or until patients’ death.

### Morbidity and mortality

Major postoperative complications were defined as delayed gastric emptying (DGE), pancreatic fistula, intraabdominal abscess, hemorrhage, reoperation and pneumonia. Pancreatic fistula, DGE and intraabdominal hemorrhage were defined according to the International Study Group of Pancreatic Surgery (ISGPS) [21-23]. Postoperative mortality was defined as death within 30 days of surgery.

### Statistical analysis

Overall survival was measured from the date of nephrectomy to the date of last follow-up or death. SAS software (Release 9.1, SAS Institute, Inc., Cary, NC, USA) was used for statistical analysis. Quantitative variables are expressed as median and range. Overall survival from the date of pancreas operation was calculated by the Kaplan-Meier estimate. Two-sided P-values were always computed and a difference was considered statistically significant at P ≤ 0.05.

## Results

### Clinicopathological features

Among the 1178 patients with PDAC or cystic tumor of the pancreas, 16 (1.35%) were also diagnosed with either synchronous or metachronous renal tumor. The patients ranged on age from 52 to 77 years (median 69 years), and showed a male predominance with 12 men and 4 women. There were 10 metachronous and 6 synchronous double tumor patients. Among these 6 synchronous cases, 5 were interdisciplinarilly resected at the same time by an urologist and a surgeon through standard median laparotomy. By the time pancreatic tumor was diagnosed, patients had received preceding nephrectomies for renal tumors 1.25 - 205 months before (median: 78.0 months). Histologically all renal tumors were RCC variants and 12 of the 16 pancreatic tumors were PDAC and the remaining 4 were intraductal papillary mucinous neoplasms (IPMN) (Table 1). Demographic, clinical and operative data, including age, sex, and the different types of renal and pancreatic resection for the entire cohort of 16 patients are given in Table 1.

**Table 1:** Patient characteristics and surgical procedures

**Case no.**	**Age**	**Sex**	**Diagnosis**	**Procedure for pancreatic tumor**	**Renal tumor**	**Procedure**	**Interval (months)**	**Phase**	**Follow-up (months)**	**outcome**
1	70	M	PDAC	ppWhipple	Clear cell RCC	Partial neph.	0	Synchronous	35	Alive
2	72	W	PDAC	ppWhipple	Clear cell RCC	nephrectomy	94	Metachronous	14	Dead
3	70	M	PDAC	ppWhipple	Clear cell RCC	Partial neph.	0	Synchronous	5.25	Dead
4	68	M	PDAC	Double bypass	RCC	Nephrectomy	119	Metachronous	4	Dead
5	77	W	PDAC	Total pancreatectomy	RCC	Nephrectomy	152	Metachronous	12.25	Dead
6	68	M	PDAC	Double bypass	RCC	Partial neph.	205	Metachronous	13	Dead
7	77	M	PDAC	Exploration	RCC	Nephrectomy	94	Metachronous	2.25	Dead
8	71	M	PDAC	ppWhippe	RCC	Partial neph.	84	Metachronous	12.5	Dead
9	71	M	PDAC	ppWhipple	Pap. RCC	nephrectomy	0	Synchronous	3.25	Dead
10	68	M	PDAC	Double bypass	RCC	Partial neph.	135	Metachronous	0	Dead
11	55	W	PDAC	Left resection	RCC	Nephrectomy	135	Metachronous	17	Alive
12	52	M	IPMN	ppWhipple	Cystic RCC	Partial neph.	0	Synchronous	8	Alive
13	65	W	IPMN	ppWhipple	RCC	Nephrectomy	3	Synchronous	27.75	Dead
14	71	M	IPMN	Total pancreatectomy	Pap. RCC	Partial neph.	1.25	Synchronous	14	Alive
15	73	M	IPMN	Total pancreatectomy	RCC	Partial neph.	205	Metachronous	32	Alive
16	75	M	PDAC	Gastroenterostomy	Pap. RCC	nephrectomy	21	Metachronous	1.5	Dead

### Treatment and prognosis

All 16 patients underwent either explorative laparotomy or pancreatic resection for pancreatic tumors. The treatment modalities for the pancreatic tumor patients included: distal pancreatectomy in 1 patient, total pancreatectomy in 3, pylorus-preserving pancreatoduodenectomy in 7, palliative bypass operation in 4 patients, and operation finished as an explorative laparotomy due to locally advanced tumor or distant mestastases in 1 patient.

Surgical complications occurred in 4 patients (25%). The most common postoperative complication was DGE (n = 2). Other complications included 1 intra-abdominal hemorrhage Grade B and 1 pancreatic fistulas (1 Grade A). Re-operation was necessary in 2 patients (12.5%) after median 11 (range 1–30) days. The median hospital stay for all patients was 25.6 (range 7 – 167) days.

**Figure 1:**
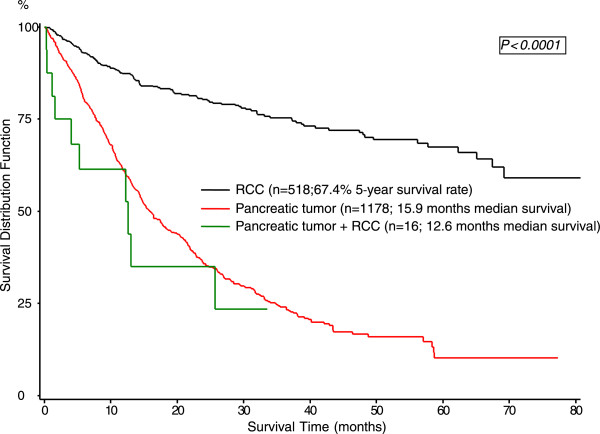
Overall survival of RCC and pancreatic tumors.

**Figure 2:**
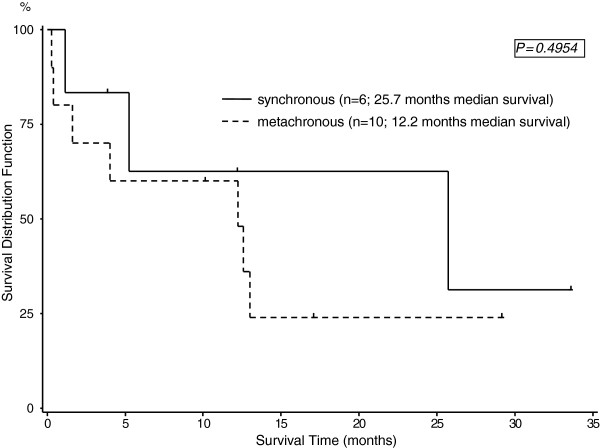
Overall survival of synchronous versus metachronous pancreatic tumor resection.

The median follow-up duration was 12.6 months with a range of 1.5-35 months. During the follow-up period 11 patients died because of either pancreatic cancer or its recurrence, whereas no patient died due to recurrent RCC. Five patients are alive at present time. Figure 1 shows the comparison of the median survival rates between the patients treated for RCC alone (n = 518), the corresponding pancreatic tumor cohort (n = 1178) with either PDAC or IPMN and the 16 patients with double primary tumors (p < 0.0001). Although lacking statistical significance, synchronous resection had a longer median survival of 25.7 months compared to metachronous resection (12.2 months) (p = 0.49; Figure 2).

## Discussion

Due to the aid of radiologic diagnostic tools, mass screening, increasing patient age [2] and improving survival rates for patients with neoplastic disease, there has been an increase in the rate of detected synchronous and metachronous second primary cancers. Although the mechanisms involved in the development of multiple primary cancers are not fully understood, several factors have been implicated. These findings are complex, and include environmental factors (tobacco and alcohol abuse, occupation, pollution), genetic predisposition, previous medical treatment (radio- or chemotherapy), gender-specific factors, hormonal factors, and interaction of these factors [24]. Studies examining genetic factors found that microsatellite instability was more frequent observed in multiple primary cancers than in sporadic cancers [25]. The incidence of double primary cancer has been carried out by the review of cancer registries in several countries, and range from 1.0 to 20% [4]. The first single case report of double cancer involving pancreatic and renal cell carcinoma was reported by Sasaki et al. in 1969 [26]. Since then, only a few more case reports have been published of this coincidence with the largest group including 6 patients. Alexakis et al. presented 2 patients with RCC and synchronous primary PDAC [8].

Several factors may account for variations in the incidence of pancreatic cancer between different countries. Since advanced age is an important factor related to pancreatic cancer, it can be anticipated that the large proportion of elderly people in the Western countries largely accounts for the high incidence of PDAC in these countries [16]. The overall reported incidence of PDAC associated with other organ malignancies is 1% to 20% [4]. Kamisawa et al. found that pancreatic cancer was associated with a high incidence of malignancies of the gastrointestinal tract, especially the stomach [27]. Gerdes et al. investigated 69 patients with PDAC and found 13 patients (19%) suffering from second primary malignancies [5], but none had RCC. A study of pancreatic cancer found 134 (5.6%) associated malignancies in 2394 autopsies including 7 with RCC (0.29%) [4]. Rabbani et al. identified multiple primary malignancies in 27% of 763 RCC patients and found an increased incidence of prostate, bladder and colorectal cancer and Non-Hodgkin’s lymphoma but no PDAC [10].

Several hereditary cancer syndromes coincide with an increased risk of pancreatic cancer. PDAC is seen in some breast cancer families with BRCA1 and BRCA2 mutations [18]. Affected family members of the FAMMM as well as those with a positive family history of ataxia-telangiectasia have much higher risk of developing PDAC compared with the general population [19]. Patients with HNPCC have an increased risk of pancreatic cancer as well as stomach, breast, small bowel, endometrial, and renal pelvis cancer [20]. None of the histories of the patients in the current study indicated any one of these rare syndromes.

Patients with a RCC have a significantly higher risk of other subsequent primary malignancies [6,24]. While prevalence studies based on autopsy series have identified a 30% to 40% incidence of other primary malignancies in RCC patients, cohort studies have identified rates of 4.5 to 27.4% [6,7]. A study by Czene et al. clearly indicates that patients with RCC are at increased risk of other cancers not only the first year after primary diagnosis, but also after more than 10 years [28]. Regarding second primary pancreatic cancers in RCC patients, environmental factors, such as dietry habits or tobacco use, and genetic factors have also been suggested to be risk factors [29]. Although this study did not examine the risk factors for both primary cancers patients, tobacco cigarette smoking is a common environmental risk factor of both cancers, approximating to a 2-fold relative risk [30]. VHL disease is a familial multiple-cancer syndrome characterized by numerous cystic and solid neoplasms [12]. More than one tumor in the brain or eye, or a single tumor in the brain or eye plus one elsewhere in the body, such as in the pancreas, kidney, liver, or adrenal gland are chraracteristic to diagnose VHL. Pancreatic cysts, serous cystadenomas and the more serious pancreatic neuroendocrine tumors but not PDAC arise in patients with VHL [12]. Nevertheless, we believe that there could be a new association between these two primary tumors. Further analytic epidemiological studies, including evaluation of gene-environment interactions, are needed to specifically identify reasons for double pancreatic-kidney tumors.

This reported “novel coincidence” of synchronous or metachronous renal cell carcinoma and pancreatic tumors in our study may be due to changes in modern medicine over time. Radiological diagnostic tools were considerably developed and diagnostic accuracy improved over the intervening period. It may be also attributable to biases of the specialization of each institution, referral patterns, and local environmental factors. The specialized nature of a cancer center like our institution allows more accurate diagnosis of primary and secondary cancers, in addition to more detailed information on staging and follow-up.

Considering the trend that the peak age of cancer patients and the incidence of cancer are increasing, the present study cautions that physicians, urologists and surgeons should consider the appearance of synchronous or metachronous pancreatic tumors in RCC patients. Because patients diagnosed with pancreatic cancer have a relatively short survival time, second primary cancer is rarely detected in these patients. Therefore, the prognosis of patients with double cancers including pancreatic cancer mainly depends on the prognosis of the pancreatic malignancy.

## Competing interest

The author(s) declare that they have no competing interests

## Authors’ contribution

SAM, MNW, and BMS had the study idea and wrote the manuscript. SP, and AH collected urological data. UH performed statistics. DJM, TH, and CL carried out the analyses, MWB and BMS edited the paper. All authors read and approved the final manuscript.
